# Statins in Asthma: Potential Beneficial Effects and Limitations

**DOI:** 10.1155/2015/835204

**Published:** 2015-11-05

**Authors:** Dipanjan Bhattacharjee, Bharti Chogtu, Rahul Magazine

**Affiliations:** ^1^Department of Pharmacology, Kasturba Medical College, Manipal University, Manipal 576104, India; ^2^Department of Pulmonary Medicine, Kasturba Medical College, Manipal University, Manipal 576104, India

## Abstract

Asthma's sustenance as a global pandemic, across centuries, can be attributed to the lack of an understanding of its workings and the inability of the existing treatment modalities to provide a long lasting cure without major adverse effects. The discovery of statins boosted by a better comprehension of the pathophysiology of asthma in the past few decades has opened up a potentially alternative line of treatment that promises to be a big boon for the asthmatics globally. However, the initial excellent results from the preclinical and animal studies have not borne the results in clinical trials that the scientific world was hoping for. In light of this, this review analyzes the ways by which statins could benefit in asthma via their pleiotropic anti-inflammatory properties and explain some of the queries raised in the previous studies and provide recommendations for future studies in this field.

## 1. Introduction

It was in July 1973 that Endo isolated compactin (ML-236B) [1], the pioneering molecule in the statin series. Almost four decades on, statins' anti-inflammatory and lipid lowering properties have found worldwide approval in prevention of cardiovascular diseases [2]. The pleiotropic anti-inflammatory properties of statins have often encouraged a plethora of studies, prodding and probing and examining its efficacy among a wide spectrum of diseases.

Asthma happens to be one such field, where prolific research with statins has exhibited significant potential in its initial stages. This new found potential application of statins assumes immense significance in today's global health scenario. Asthma is a massive global health problem, afflicting people across the spectrum, irrespective of age groups or social bearings. The earliest references to asthma in the annals of history can be traced back to the ancient Chinese and Egyptian civilizations [3]. The term “*asthma*” has its roots in the Greek word “*aazein*,” which literally translates into “to pant” [4]. The scientists have tried to gain an insight into the pathophysiology of asthma across generations and civilizations. In 1886, Bosworth's revolutionary work proposed an underlying allergic mechanism for asthma [5]. This opened up a new avenue for exploration and obtaining a better understanding of asthma. Despite this revolutionary breakthrough, the status of asthma precariously swung from that of an allergic condition to being a psychiatric illness as was the case in the 1930s to the 50s when it was one of the “*holy seven*” psychosomatic illnesses [6]. Asthma was not established as an inflammatory disorder until the 1960s, when use of corticosteroids attained the status of first line of treatment for asthma. Thenceforth, many drugs, bearing different target sites in the asthma pathophysiology have been introduced.

Inspite of the rapid strides being taken in the treatment of asthma, it still poses a huge burden on the society, both medically and economically. Recent WHO estimates peg the global asthma sufferers' number at 100–150 million people, a number equivalent to the populace of the Russian Federation [7]. The annual mortality numbers rack up to 180,000 globally [7]. India has 15–20 million asthmatics, roughly 1.6% of the whole country's population [7]. The financial burden posed globally by asthma far exceeds the costs incurred by tuberculosis and HIV (Human Immunodeficiency Virus)/AIDS (Acquired Immunodeficiency Syndrome), put together [7]. These mounting figures point at the lacunae existing between the existing therapies and the desired outcome in treatment of asthma.

Asthma being a chronic disease, by virtue of multiple assaults, resulting from contact with allergens or various triggers, produces chronic airway inflammation and remodeling [8]. The chronic inflammation and structural alterations lead to airflow limitation and decrease in airway caliber, leading to increased morbidity and mortality. Despite the existence of a few therapies to alleviate this seemingly chronically developing component of asthma pathophysiology, their efficacy has often been questionable especially in terms of the side effects arising due to long-term usage. Hence, this has sparked off a flurry of studies, aimed at developing molecules that might help mitigate the chronic inflammation and airway structural changes effectively, thus helping in filling the existing void in the treatment of asthma. Of the many molecules that have been tinkered with, statins in their initial stages emerged as potentially suitable group of drugs. However, recent evidences show that statins seem to be losing ground as potential antiasthma drugs. In the subsequent sections in this review, an attempt is made to decipher the mechanism(s) by which statins exert their anti-inflammatory effects, by virtue of which they may fulfill their potential as drugs against asthma in the future. Additionally, we have tried to examine the possible reasons that may have contributed to the inconsistent results evidenced in various clinical studies examining the role of statins in asthma to date. Further, certain recommendations have been put forth for the future clinical studies.

## 2. Preclinical Studies

Statins have emerged as a multifaceted series of drugs. The anti-inflammatory effects of statins are well documented in the cardiovascular literature. However, the actions of statins on the respiratory tract are still unclear. The evidences emerging from multiple preclinical and animal studies have been quite encouraging. The possible mechanisms by which statins may benefit in asthmatics have been summarized as follows.

*Mitigatory Effects of Statins on Asthma Pathogenesis*

 Countering airway remodeling by
  - mitigating airway epithelial changes [9, 10],
  - checking subepithelial fibrosis [11, 12],
  - inhibitory influence on the contractile regulatory proteins [13, 14].
 Reduced airway inflammation due to a 3-fold action:
  - role of nitric oxide [15–17],
  - decreased inflammatory cytokine production [11, 18],
  - reduced airway inflammatory cells influx [19, 20].

To obtain a better insight into the role of statins in asthma, the different mechanisms are discussed below.

### 2.1. Airway Remodeling

Until two decades ago, airway inflammation had been the center of attention for researchers investigating asthma pathogenesis. However, in the last 2 decades, airway remodeling has garnered a significant proportion of the “scientific” eyeballs [21]. First described in 1922 by Hubert and Koessler, airway remodeling can loosely be defined as the structural changes in asthmatics' airways that are absent among normal individuals [22]. It includes a spectrum of alterations to the airway architecture ranging within loss of epithelial integrity, thickening of basement membrane, subepithelial fibrosis, goblet cell hyperplasia, airway smooth muscle cell hypertrophy and hyperplasia, loss of integrity of cartilaginous structures, and neovascularization [22]. It has been observed in various preclinical and animal studies that statins may exert beneficial influences against a few of the phenotypic changes seen in asthmatics' airways during the course of airway restructuring.

Goblet cell hyperplasia, a structural hallmark of airway epithelium in asthmatics, is associated with increased expression of MUC5AC protein, which in turn is responsible for increased mucin production. Studies have shown that treatment with statins causes decreased MUC5AC gene expression, leading to decreased mucin production and suppression of goblet cell hyperplasia [18, 23]. Further, another study performed in animal models revealed inhibition of goblet cell hyperplasia, which was hypothesized to be due to inhibition of the mevalonate pathway [9]. The reduction in goblet cell hyperplasia subsequently leads to decreased mucin secretion in airways and reduction in airflow limitation. Besides goblet cell hyperplasia, there are a few studies that have tried to examine the effect of statins on the other structural changes seen in airway epithelium in asthmatics. One such study concluded that simvastatin could protect the airway epithelium from tobacco smoke induced airway epithelium denudation [10]. In a separate paper, the authors have postulated that the cytoprotective effects of statins could be due to the induction of proresolution mediators like 15-epi-lipoxin A_4_ (15-epi-LXA_4_) [24]. Another separate study showed that statins could modulate the expression of proinflammatory cytokines and chemokines in the mouse airway epithelium and thus alleviate the airway epithelial inflammation and structural remodeling [25, 26].

A very critical tenet of airway remodeling, that is, subepithelial fibrosis and thickening of basement membrane, occurs due to an imbalance between extracellular matrix (ECM) deposition and degradation, with the balance tilted in favor of deposition [27–29]. There occurs a significant amount of proteoglycan deposition in the lamina reticularis layer, located just beneath the basement membrane resulting in its thickening. This is brought about by the secretion of various cellular components like neutrophils, interstitial cells, and macrophages [22]. Among the major groups of proteases that are secreted by these cells, matrix metalloproteinases (MMPs) are the most prominent [22, 30]. Further, MMPs have been implicated in airway remodeling due to smooth muscle hypertrophy [31] and angiogenesis [32]. Statins have been shown to decrease the expression of the metalloproteinases, especially MMP-9 [11], and thus may mitigate the subepithelial fibrosis and thickening of the basement membrane. Another study further substantiated the role played by statins and geranylgeranyl transferase inhibitors (GGTIs) in inhibiting the synthesis and deposition of extracellular matrix by the airway smooth muscle cells. The authors have suggested that it could be due to the suppression of geranylgeranyl transferase 1 (GGTase 1) mediated posttranslational modification of the signaling molecules like RhoA [12]. Geranylgeranyl transferase 1 enzyme, which is a crucial enzyme for posttranslational modifications of Rho family GTPases, has been implicated in influencing the autophagy and apoptosis in airway smooth muscle cells [33]. Inhibitors of mevalonate pathway like statins and GGTase 1 could produce apoptosis and autophagy of the airway smooth muscle cells and may contribute to airway remodeling [33].

**Figure 1:**
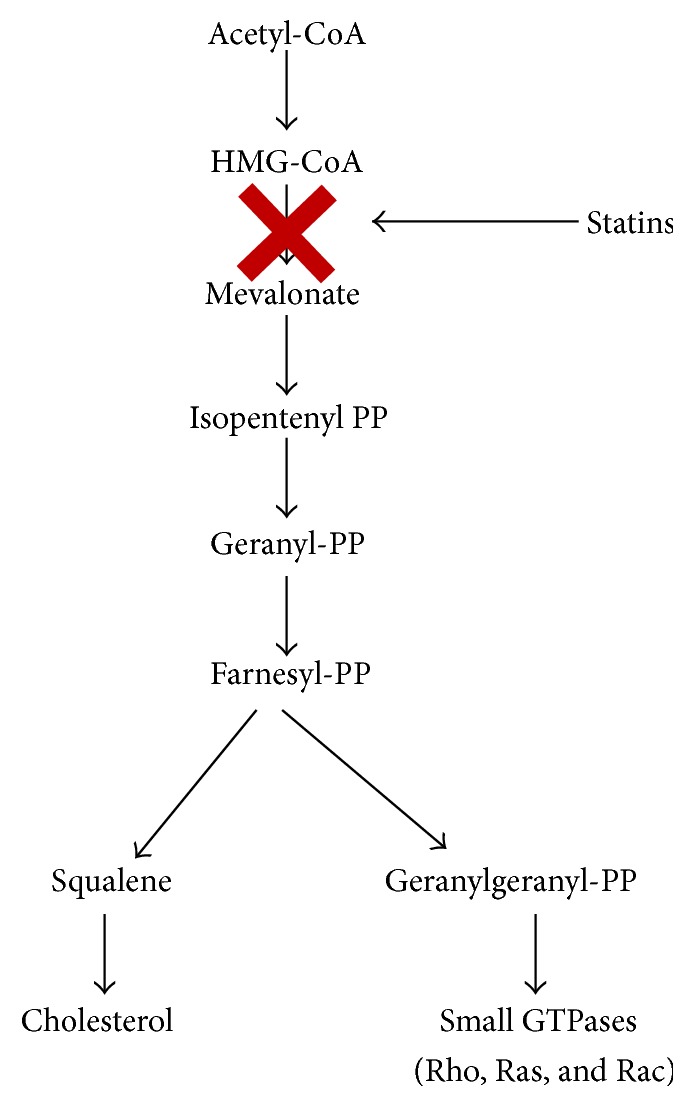
Effect of statins on the mevalonate pathway.

Among the various components of airway remodeling, statins inhibitory influence over the contractile regulatory proteins in the airway smooth muscles has been studied in great detail. Statins, via inhibition of the rate limiting enzyme HMG CoA reductase, prevent the synthesis of L-mevalonate. Subsequently, this prevents the formation of the downstream intermediates of the mevalonate pathway (shown in Figure 1), for instance, geranyl pyrophosphate (GP), farnesyl pyrophosphate (FP), and geranylgeranyl pyrophosphate (GGP) [34]. These molecules are termed as the “isoprenoid” derivatives, which are responsible for posttranslational modifications of proteins by the covalent attachment of farnesyl or geranylgeranyl groups to cysteine residues via the formation of a thioether linkage at the C-terminal in the peptide chain. The C-terminal is constituted structurally as CaaX, where “C” represents the cysteine amino acid, “a” represents aliphatic amino acid, and “X” represents any amino acid. Farnesylated proteins have alanine, methionine, or serine amino acids in place of “X” and geranylgeranylated proteins have leucine amino acid in place of “X.” Once the prenyl group attaches to the cysteine residues, the –aaX segment is proteolytically cleaved, thus exposing the carboxyl group which in turn gets esterified by the attachment of a methyl group from a methyl donor molecule like S-adenosyl methionine. This entire process is termed as “prenylation” [35].

The process of prenylation assumes immense significance as the hydrophobic prenyl group is critical for the anchoring of extracellular signaling proteins to cell membranes, which subsequently can interact with various specific receptors on the cellular membranes to trigger various intracellular signaling pathways including those mediated by small guanosine triphosphate (GTP) binding proteins belonging to the Ras GTPases superfamily [36]. This superfamily can be further broken down into small families of which Ras, Rho, Rab, Ran, and Arf are the major ones [37]. The activation of these small GTPases, like Rho, Rac, and Ras, is also guided by their isoprenylation status.

Among the three Ras families of small GTPases, Rho plays a critical role in airway remodeling and hyperresponsiveness. The Rho family of small GTPases controls airway smooth muscle contraction via their action overregulatory contractile proteins. Myosin light chain is one of the major airway contractile regulatory proteins [38]. Its activated/phosphorylated state brought about by myosin light chain kinase (MLCK) is responsible for the contraction of smooth muscles, whereas myosin light chain phosphatase (MLCP) is responsible for dephosphorylation of myosin light chain which in turn leads to relaxation of airways. When Rho kinase, a downstream effector molecule of Rho based signaling pathway, is induced, it causes phosphorylation of the myosin binding subunit of MLCP. This, in turn, indirectly leads to an increase in myosin light chain phosphorylation and an enhanced state of airway smooth muscle contraction [39]. It has also been hypothesized that Rho kinase can directly phosphorylate the myosin light chain at* ser*-19 residue, which is the site at which MLCK acts upon [40]. Evidences have emerged to show that Rho kinase can also phosphorylate CPI-17, which in turn causes inhibition of MLCP activity [41]. Additionally, it has been conjectured that Rho kinase mediated phosphorylation of calponin may also contribute to airway smooth muscle contraction [42].

Besides the effect on various contractile regulatory proteins, evidences from studies indicate the central role of RhoA pathway in regulating actin cytoskeletal dynamics, which is responsible for determining the force and the shortening of airway smooth muscle (ASM) cells [43, 44]. In addition, the theory of pathophysiology primed Rho kinase mediated calcium sensitization leading to increased smooth muscle contraction as seen in cardiovascular diseases can also be applied to airway diseases as seen by the results of a new study [45]. The increased expression of RhoA and Rho kinase in the bronchial smooth muscle cells following repetitive allergen challenges as seen in various animal studies points to the potentially critical role of Rho kinase in the magnitude and possibly the development of airway hyperresponsiveness [45–48].

Recent studies have indicated a role of RhoA and Rho kinase at a genetic level. It has been evidenced that RhoA pathway may be essential for transcription of smooth muscle genes in the airways [49–54]. Further, the airway thickening brought about by the fibroblasts and ASMs may be attributed to the Rho mediated control of the transcription factors like NF-*κβ* and activator protein-1 (AP-1), albeit the degree of activation and the extent of contribution might be influenced by the presence of the G-protein coupled receptor agonists [55–57]. It has also been hypothesized that Rho kinase may be implicated in the regulation of the ASM and fibroblast migration [58, 59].

Besides Rho, Ras protein also plays a significant role in smooth muscle proliferation and hypertrophy [60–63]. As seen in the asthmatics, there is upregulation of various inflammatory mediators like platelet derived growth factor (PDGF) and endothelial growth factor (EGF) whose activity gets augmented due to an overexpression of growth factor receptors with intrinsic tyrosine kinase activity as well as various G-protein coupled receptors. Subsequently due to the activity of these mediators, p21ras activation occurs, which in turn sparks off two signaling pathways, that is, extracellular signal-regulated kinase (ERK) and phosphatidyl-inositol-3-kinase (PI-3-K) pathways. ERK pathway leads to induction of deoxyribonucleotide (DNA) synthesis and cellular proliferation. PI-3-K pathway induces cyclin D1 production, which in turn leads to cellular proliferation. By inhibiting the synthesis of the isoprenoid derivatives, prenylation of the small GTPases proteins can be interfered with. This in turn can lead to the mitigation of airway smooth muscle hypertrophy and hyperplasia as evidenced by few studies [13, 14].

### 2.2. The Key to Countering Airway Inflammation

Statins “pleiotropic” anti-inflammatory property has often propelled the research into utility against asthma. Since the past few decades, tremendous controversy has raged on as regards the role of nitric oxide (NO) in the pathophysiology of asthma. It has been observed that nitric oxide exhibits both beneficial and detrimental influences over asthma pathology. Nitric oxide is produced by nitric oxide synthase (NOS) during the conversion of L-arginine to L-citrulline. NOS exists in three different isoforms, that is, two constitutive (types I and III) and one inducible forms (type II) [64].

Nitric oxide so produced by the constitutive isoforms, that is, neuronal NOS (nNOS) and endothelial NOS (eNOS), induces cGMP (cyclic guanosine mononucleotide phosphate) production, which in turn produces vasodilatation and possibly bronchodilatation [64, 65]. Many animal studies [66–69] have shown that exogenously administered nitric oxide can act as a potent dilator of tracheal and airway smooth muscles, especially of the proximal airways, thus pointing to a potential utility of nitric oxide donors or agonists as therapeutic options against asthma. The effect over the distal airways is still under a shadow, though. However, as per the results from various studies, it is believed that vasodilatory properties of nitric oxide, especially under the influence of inducible NOS (iNOS), can lead to extravasation of plasma, subsequently producing edema of the airways and increased mucus production and further worsening the bronchoconstriction [70–72]. To compound the ambiguity over the role of NO in asthma pathogenesis, observations from few studies have also revealed that NO produced by the airway epithelium may exert positive influence over the mucociliary clearance [73–75].

It has been observed from various studies that iNOS may be proinflammatory [76–79] as it has been shown to be responsible for the recruitment of neutrophils, eosinophils, and other inflammatory cells and production of various inflammatory cytokines. The ability of statins in countering the proinflammatory iNOS has been studied as furnished by the evidences from few studies [15, 16]. The authors have postulated that statin mediated inhibitory activity on iNOS may be seen as a result of increase in the levels of I*κβ* (inhibitor of NF-*κβ*) as well as by the inhibition of IFN-*γ* induced STAT1 (signal transducer and activator of transcription 1) phosphorylation, which might be induced via genetic modulation.

On the other hand, the eNOS levels have been shown to be augmented by the action of statins [17]. In this study, it was seen that simvastatin reduced the iNOS but increased eNOS levels. Along with the NOS levels, the levels of nitrotyrosine free radical levels were decreased, possibly due to decrease in iNOS levels. eNOS derived NO has been shown to inhibit airway inflammation by inhibiting the expression of NF-*κβ* [80–84] and subsequently mitigating the expression of iNOS and release of inflammatory cytokines. The phenomenon of inhibition of iNOS by eNOS assumes significance in the light of the fact that statins have been shown to increase the expression of eNOS massively. It has been postulated that the statin mediated increase in NO production, especially via eNOS, may exert beneficial influence on asthmatics. There are many evidences from various animal models to support the fact that endogenous or constitutive nitric oxide may play a modulatory role in airway hyperreactivity [85–87]. It was observed in these studies that the bronchodilatation following application of bradykinin, endothelin-1, substance P, and calcitonin gene related peptide in the tracheal tube preparations, was reversed into bronchoconstriction after administration of NOS inhibitors. It is important to bear in mind that the NOS under the spotlight is the endogenous or constitutive NOS, among which eNOS forms the major chunk. The results obtained from the animal models, on extrapolation to clinical settings, yielded similar results [88]. Hence the authors have suggested that endogenous nitric oxide may play a bronchoprotective role in mild asthma. However, the results of another clinical RCT showed that the NOS inhibition of airway hyperreactivity to bradykinin in severe asthmatics was muted [89]. This was attributed to probably a decrease in the proportion of endogenous NOS in severe asthmatics. In another clinical trial, high doses of corticosteroids were used in severe asthmatics and it was found that there was increased airway hyperreactivity to bradykinin following NOS inhibitor administration [90]. This was explained by the authors with a hypothesis that corticosteroids suppress the production of iNOS and thus increase the sensitivity to the endogenous NO levels. Those studies conducted in both animal and clinical settings attest to the protective role of endogenous NOS in the airways.

Statins can augment the levels of eNOS by inhibition of Rho/Rho kinase producing a massive increase in the endothelial nitric oxide synthase (eNOS) since Rho inhibits endothelial nitric oxide (NO) generation [91]. Further, recent studies have illustrated that statins cause activation of protein kinase Akt, which in turn leads to increase in eNOS phosphorylation and subsequently increased NO generation [91]. These mechanisms for rapid generation of NO exist besides the statin induced upregulation of eNOS gene expression [92]. Statins also decrease the expression of caveolin-1 in the endothelial cells, responsible for subcellular localization of eNOS and its inactivation [93].

Studies have also elicited another unique anti-inflammatory association of statins with eNOS. ADMA (asymmetrical dimethyl arginine) mediates endothelial dysfunction by facilitating uncoupling of eNOS [94]. Coupling refers to the enzymatic state where metabolism of L-arginine and shuttling of electrons are coupled with NO production instead of oxygen. It has been observed that statins positively modulate eNOS coupling by upregulating the dimethyl arginine dimethyl amino hydrolase (enzyme for metabolism of/ADMA) gene transcription and in turn promoting the metabolism of ADMA [95]. Thus statins, by inducing a massive increase in eNOS and subsequently NO production, may potentially stand to negate the inflammatory phenomena in asthma.

Besides the effect on NO, it has been observed that statins can induce endothelial cell repair and regeneration by inducing the expression of vascular endothelial growth factors (VEGF) [96] and increasing the circulatory endothelial progenitor cells [97]. Statin induced modification of redox states in the vascular endothelium leads to suppression of redox sensitive pathways that are responsible for modulating the expression of many proinflammatory genes [98]. Statins have been shown to attenuate the release of proinflammatory cytokines and chemokines from the vascular smooth muscle cells (VSMCs), for instance, matrix metalloproteinase-9 (MMP-9) [11] and various intracellular pathways like Rho kinase and MAPK [99], thus inhibiting extracellular remodeling. The underlying mechanism hypothesized involves statin induced reversible inhibition of prenylation of GGP mediated Rho kinase and DNA synthesis inhibition [100]. Further, it has been noted that inflamed respiratory epithelial cells release various inflammatory cytokines, for instance, CCL11, CCL24, and IL-6 [101]. CCL11 and CCL24 are eosinophil chemoattractants (eotaxins), whereas IL-6 induces lung injury and fibrosis [18]. It is believed that the eosinophils migrate to the inflammation site and adhere to the concerned inflamed cells by virtue of proteins like ICAM-1 (intracellular adhesion molecule-1) [102]. Subsequently, eosinophils induce tissue damage by secreting major basic protein, eosinophil cationic protein, eosinophil peroxidase, and eosinophil derived neurotoxin [103]. Animal studies have emphatically illustrated that statin treatment decreases the eosinophil migration into the inflamed tissue by decreasing the production of the chemoattractant molecules and by decreasing the expression of ICAM-1 gene in lung tissues [18]. Further, it has been shown that statins decrease the production of IL-6 in the inflamed airway tissues and thus reduce damage to the lungs by fibrosis [18].

Further, asthma is characterized by the release of various proinflammatory cytokines from T helper type 2 cells, for instance, IL-4, IL-5, IL-6, IL-13, and IL-17 and TNF-*α*, that contribute to airway inflammation via different mechanisms [104]. Secretion of IL-4 leads to B-cell activation and increases the production of IgE and IgG [105]. IgE specifically is involved with the mast cell activation, which in turn produces the features of allergic inflammation [106]. IL-5 stimulates eosinophils production from bone marrow [107]. IL-13 has been shown to be associated with isotype switching of antibodies to IgE [104]. IL-17 and TNF-*α* secretion, if lowered, can lead to suppression of airway inflammation and amelioration of airway remodeling. Thus, evidences furnished by the preclinical animal studies repeatedly point to the fact that statin usage may cause a reduction in the expression of the major proinflammatory cytokines [19, 20], which in turn could also suppress the recruitment of various inflammatory cells. This can lead to the suppression of airway inflammation and amelioration of respiratory symptoms and mitigation of the disease pathology.

## 3. Clinical Trials

The positive results derived from the preclinical studies fuelled a flurry of clinical studies over the past decade (summarized in Tables 1 and 2), aimed solely at investigating the effect of statins in asthma. Overall these results are not consistent and there is a need for further studies and discussing various issues risen up following these clinical studies carried out in the past.

### 3.1. Can Orally Consumed Statins Reach the Airway and Attain Sufficient Concentration to Exert Their Pleiotropic Properties [121]?

In clinical trials [108–110, 112–120, 122–124] carried out to date, statins have been administered by oral route alone, which is the only approved route of administration in humans. Most of the statins, once absorbed into the systemic circulation, are extensively metabolized in the liver. This raises questions about the ability of orally administered statins to reach the respiratory compartment at therapeutic concentrations. Further, it raises the issue whether inhalational route of statin administration would be better suited to counter asthma than oral route. There have been a few animal studies that have reported better results with inhalational routes of administration of statins as compared to other systemic routes [125, 126]. However, there are no published reports of other routes in humans. This can be best ascertained by various pharmacokinetic and pharmacodynamic studies aimed at quantifying the proportion of statins reaching the respiratory system. The nonavailability of a lung biomarker or a genetic biomarker to indicate the accumulation of statins at therapeutic concentration in the respiratory system further compounds the issue.

### 3.2. Are We Using the Right Dose and Type of Statins?

An extensive scrutiny of various randomized controlled clinical trials (RCTs) and retrospective studies revealed variations in the doses of statins so used. The most common reasons for choosing the concerned dose of statin in various trials are as follows.

- The clinical benefit seen in previous trials examining statins in rheumatoid arthritis at the same dose (40 mg once a day) [112, 114].
- The dose (40 mg once a day) being greater than the ones used in previous failed studies [114].

One can find many arguments against the line of thinking behind the reasons ascertained here. Asthma is a chronic inflammatory condition. For statins to be effective as an anti-inflammatory agent in asthma, they have to be administered at doses which can counter the active inflammatory processes. Though, in daily practice, doses equivalent to or lower than 40 mg are the most commonly preferred doses in patients with cardiovascular diseases, recent guidelines for cardiovascular event management suggest that for secondary prevention treatment should be initiated at the highest tolerated dose possible (e.g., 80 mg for atorvastatin) [127]. This is done not only to counter the highly deranged lipid profile, but also to counter the active inflammatory processes in the atherosclerotic plaques in the vascular system in such patients. Thus, the success achieved with high dose statins in cardiovascular diseases and the mixed results obtained using low dose statins in asthma lends credence to the premise that high dose statins (e.g., atorvastatin 80 mg) may exhibit better results at countering the acute inflammatory stage in asthma. It is our belief that once the acute inflammation is taken care of, statins can be tapered off to a more moderate dose for maintenance purposes in those asthma patients who start exhibiting toxicity to statins.

Further, the pathophysiology of asthma and rheumatoid arthritis are quite different from one another. Also, there exists ambiguity over what could be the relative effects of statins in various compartments of the body, that is, the vascular airway and the musculoskeletal compartments. This in turn might be responsible for variations in the actions of statins against various diseases. Additionally, the question arises as to whether the mucosal epithelial immunity and alveolar capillary barriers in the respiratory compartment interfere with the action of statins [121].

Another factor responsible for the inconsistent results could be the different types of statins that have been used across various studies. Most commonly used statin was atorvastatin [112, 114, 116, 117, 119] which is less lipophilic as compared to simvastatin [113, 115] that was used in a few other clinical studies. The chemical structure of statin bears a critical relation with its activity. The structural differences lead to significant differences in the affinity for the active site of HMG CoA reductase enzyme, entry into hepatic and nonhepatic tissues, bioavailability, and modes of biotransformation and excretion. For instance, one study conducted on human smooth muscles showed that lovastatin and simvastatin exhibited higher sensitization to apoptotic agents as compared to atorvastatin, whereas pravastatin exhibited no effect at all [128]. The results of another study exhibited that lipophilic statins may exert a greater anti-inflammatory influence on cytokine production by human monocytes* in vitro* and mice leucocytes* in vivo* [129]. Further, it has been observed that statins may differ in terms of protein expression [130–135]. A clinical study [136] adds substance to this claim. It was observed that atorvastatin and simvastatin showed different* ex vivo* immunological responses as atorvastatin led to downregulation of human leucocyte antigen (HLA-DR) and CD38 activation marker on peripheral T cells. On the other hand simvastatin produced a significant upregulation of these markers. The effect on the superantigen mediated T cell activation was just the opposite. So, the differences in the pharmacological properties could lead to variations in their therapeutic efficacy and development of adverse effects.

### 3.3. How Long Do Statins Take to Exert Their Effects?

As amazing the anti-inflammatory properties of statins so recorded in the cardiovascular literature might be, it is imperative to note that the vascular wall remodeling requires not weeks or months, but rather years of treatment. It is highly likely that the same temporal relationship between treatment and its effects might be applicable in case of asthma. A study performed in mice showed that the half-life of aortic smooth muscle cells division ranged between 300 [137] and 800 [138] days. By extrapolation of this data to humans, it becomes clear that the time required by statins to exert any meaningful effect on asthma pathophysiology, especially on the airway remodeling, will be in the range of years and not few weeks or months which is the study duration in most of the clinical studies [109, 110, 112–117, 119]. It could potentially be the single most important factor behind the inconclusiveness of the results of the clinical studies. Statins, if given for sufficient time, may be able to fulfill its potential as the anti-inflammatory antiasthma agent that we have been looking for.

### 3.4. Are Statins Better as Sole Agents or in a Combination Regimen?

The single most important reason for the less number of studies utilizing statins as a part of combination regimen is that the anti-inflammatory effect of statins on human airway has not yet been established beyond reproach. Hence, most of the studies that we utilized in our analysis have examined statins as a sole therapeutic agent. A few studies have evaluated the efficacy of statins as part of a combination regimen. One study revealed the synergistic action of statins with corticosteroids, thus opening up an avenue for utilizing statins along with corticosteroids, especially in case of severe eosinophilic asthma [120]. Another clinical trial compared favorably a combination of statins along with short acting beta agonist and corticosteroids with a combination of corticosteroids and short acting beta agonists in terms of lung function tests [118]. A study recently attested to the effectiveness of atorvastatin in combination with beclomethasone among smoker asthmatics [139]. Further, results from another retrospective study also yielded that statins in an adjuvant capacity may yield more benefits in controlling of asthma symptoms [111]. In view of the long duration of time required for statins to exert their effects, it seems plausible that statins can be used as a long-term maintenance therapy agent. Further, utilizing statins in combination with other drugs will reduce the required doses of the other antiasthma agents and decrease the possibilities of side effects of drugs like corticosteroids, especially in severe cases. In addition, if synergism is obtained in the mechanism of actions of statins and other agents, it would lead to increased efficacy of the treatment options and potentially lead to better control of symptoms and mitigation of the disease pathological process. Hence future study designs should take the benefits of combination therapy regimens into consideration.

### 3.5. Do Statins Exert Preferential Activity in a Particular Asthma “Phenotype”?

As per Global Initiative for Asthma (GINA), recognizable clusters of demographic, clinical, and/or pathophysiological characteristics are termed as “asthma phenotypes” [140]. These phenotypes have been classified as allergic, nonallergic, late onset, fixed airflow limitation, and obese asthmatics [140]. In the absence of any strong relationship between certain pathological characteristics and clinical paradigms or responses to treatments, there is a lack of phenotype based treatment guidelines. However, our literature search revealed a few studies which show a favorable relationship between statins and smokers [122, 123, 139]. In addition, the patient's asthma status seemed to be a significant factor in the response to statins among smokers [112]. Further, smokers exhibited reduced sensitivity to inhaled corticosteroids [124]. However, it has been observed that statins can enhance the anti-inflammatory activity of corticosteroids [120]. Thus statins may possess a possible niche amongst asthmatic smokers who have reduced responsiveness to inhaled corticosteroids. Addition of statins to ongoing treatment may help in improving the clinical and functional parameters in severe asthmatics [111, 118, 120]. But the results with other studies involving mild to moderate asthmatics [108, 110, 113, 114, 116, 117, 119] did not show statins in positive light. An* in vitro* asthma study showed that a combination of fluticasone and simvastatin augmented anti-inflammatory activity of regulatory T cells and T helper-17 (Th-17) cell in response to airway inflammation [141]. In light of this, it may be surmised that as severe asthma bears neutrophilic and Th-17 cells induced inflammation at its core, statins by altering this relevant biology in severe asthma may exhibit a greater propensity to benefit in severe cases rather than mild or moderate grades of asthma. However, it would be unwise and hasty if further studies are not conducted to verify the role of statins among mild and moderate grade of asthmatics. Additionally, varied patient characteristics should be adopted as part of the inclusion criteria in the future studies so that the full range of statin applications in asthma can be explored upon.

Recommendations suggested in the following list would go a long way in standardizing the future studies. To conclude, in view of the multifaceted nature of statins, it becomes imperative that the scientific community tries to investigate and answer these queries, so that this interesting class of drugs can be used in asthma as well.

*Recommendations for Future Studies*

1. Mechanistic study should be conducted to determine whether the accumulation of statins at therapeutic concentration occurs in respiratory system. If so, then comparative studies should be performed between inhalational and oral routes to determine which route is more efficacious.
2. Development of a stable marker (lung/systemic) should be carried out to ascertain the availability of statins at therapeutic concentration throughout the treatment.
3. Dose ranging studies should be conducted with multiple types of statins to determine the type and its optimal dose which produces maximum efficacy in asthmatics.
4. Long duration studies should be conducted keeping in mind the slow onset of anti-inflammatory activity of statins and the slow nature of airway remodeling.
5. In view of possible synergistic action between statins and other asthma drugs, instead of sole treatment regimen, combination of statins with other asthma treatment drugs like inhaled corticosteroids and long acting beta agonists should be utilized in the test subjects.
6. In order to prove the efficacy of statins as anti-inflammatory agents in asthma, severe eosinophilic asthma patients as well as smokers should be targeted. If the statins do show significant efficacy in this phenotype, then other asthma phenotypes could be investigated.

## Figures and Tables

**Table 1:** Summary of retrospective clinical trials and their results used in our review.

Study name	Study type	Statin used	Dose used (mg per day)	Study sample	Statin usage duration	Results (i) Pulmonary function results (ii) Airway and serum inflammation (iii) Asthma symptoms (iv) Acute exacerbations
Ostroukhova et al. [108]	Retrospective study	—	—	50	2 years	(i) 3% to 5% median worsening of FEV1 (ii) Not measured (iii) Not measured (iv) Maintenance medication, nocturnal awakenings, and albuterol use increased

Pagovich et al. [109]	Retrospective study	Atorvastatin, simvastatin	—	70	4 weeks	(i) Pulmonary functions improved (ii) Not measured (iii) Not measured (iv) Not measured

Adams et al. [110]	Retrospective study	—	—	539	Not mentioned	(i) No significant difference (ii) Not measured (iii) Not measured (iv) Not measured

Zeki et al. [111]	Retrospective study	Atorvastatin Simvastatin Lovastatin Pravastatin	—	165	1–5 years	(i) No significant difference (ii) No significant difference (iii) Significantly higher ACT score (iv) No significant difference

RCT: randomized controlled trial. AQLQ: Asthma Quality of Life Questionnaire.

FEV1: forced expiratory volume in 1st second. PEF: peak expiratory flow.

ACQ: Asthma Control Questionnaire. FENO: fractional exhaled nitric oxide.

**Table 2:** Summary of prospective clinical trials and their results used in our review.

Study name	Study type	Statin used	Dose used (mg per day)	Study sample	Statin usage duration	Results (i) Pulmonary function results (ii) Airway and serum inflammation (iii) Asthma symptoms (iv) Acute exacerbations
Braganza et al. [112]	RCT	Atorvastatin	40	71	4 weeks	(i) No significant difference (ii) No significant difference (iii) ACQ and AQLQ increased (iv) Not measured

Menzies et al. [113]	RCT	Simvastatin	20, 40	16	4 weeks	(i) No significant difference (ii) 0.86 geometric mean fold decrease in FENO (iii) Not measured (iv) Not measured

Hothersall et al. [114]	RCT	Atorvastatin	40	54	8 weeks	(i) No significant difference (ii) Macrophage count and sputum fluid leukotriene B4 decreased (iii) No significant difference (iv) Not measured

Cowan et al. [115]	RCT	Simvastatin	40	43	4 weeks	(i) PEF, FEV1 increased (ii) Sputum eosinophils decreased (iii) ACQ decreased (iv) Not measured

Foumani et al. [116]	RCT	Atorvastatin	40	67	8 weeks	(i) No significant difference (ii) Not measured (iii) Not measured (iv) Not measured

Fahimi et al. [117]	RCT	Atorvastatin	10	17	4 weeks	(i) No significant difference (ii) Not measured (iii) Not measured (iv) Morbidity decreased

Feschenko et al. [118]	RCT	Atorvastatin (along with ICS and salbutamol)	40	31	4 weeks	(i) Morning PEF, FEV1 increased (ii) Not measured (iii) Not measured (iv) Night symptoms, cough, daily symptoms, and use of salbutamol decreased

Moini et al. [119]	RCT	Atorvastatin	40	62	8 weeks	(i) No significant difference (ii) Not measured (iii) Not measured (iv) Not measured

Maneechotesuwan et al. [120]	RCT	Simvastatin	10	47	8 weeks	(i) No significant difference (ii) Sputum eosinophils reduced (iii) Not measured (iv) Not measured

RCT: randomized controlled trial. AQLQ: Asthma Quality of Life Questionnaire.

FEV1: forced expiratory volume in 1st second. PEF: peak expiratory flow.

ACQ: Asthma Control Questionnaire. FENO: fractional exhaled nitric oxide
